# Simulation of bi-directional pedestrian flow in corridor based on direction fuzzy visual field

**DOI:** 10.1038/s41598-023-46530-0

**Published:** 2023-11-07

**Authors:** Shiwei Li, Qianqian Li, Ganglong Zhong, Yuzhao Zhang

**Affiliations:** School of Traffic and Transportation, Lanzhou Jiaotong University, Lanzhou, 730070 China

**Keywords:** Computational science, Fluid dynamics

## Abstract

Bi-directional pedestrian flow in corridors is a complex dynamic system due to the diversity in pedestrian psychological characteristics. Incorporating individual differences of pedestrians is vital for improving pedestrian flow models. However, due to the inherent complexity and variability of pedestrian movement, model parameter calibration remains challenging. Controlled experiments are needed to collect empirical pedestrian movement data under different environments. This enriches the database on pedestrian movement patterns and provides necessary support for improving pedestrian flow models. To address this issue, we conducted controlled experiments to quantify pedestrian heterogeneity by defining the direction of fuzzy visual field (DFVF). The DFVF incorporates various static and dynamic pedestrian factors. We used it to modify the traditional cellular automata model. This improved model simulates bi-directional pedestrian movements in the corridors, reproduces density-speed and density-volume relationships, and reveals self-organization phenomena. Furthermore, an analysis was conducted to examine the impacts of pedestrian density and facility spatial layout on evacuation time. Pedestrian interactions were also studied to uncover fundamental bi-directional flow properties. As pedestrian density increased, the evacuation time showed an exponential upward trend. Corridor length significantly impacts evacuation time, while increasing corridor width helps control it. As crowd density increases, pedestrian flows exhibit three distinct steady states: the strolling flow at low densities, directional separated flows at medium densities, and dynamic multi-lane flows at high densities. In summary, the modified cellular automata model successfully incorporates pedestrian heterogeneity and reveals intrinsic bi-directional pedestrian flow patterns. This study provides valuable insights for pedestrian facility design and optimizing pedestrian flow organization.

## Introduction

Bi-directional pedestrian flow refers to the scenario where two directions of pedestrian flow move in opposite directions and conflict with each other. There are two directions of pedestrian flow. This type of scenario is very common in real life, for example, in rail transit passages, urban sidewalks or pedestrian overpasses, school corridors or staircases, etc. Due to the effect of opposite conflicts, bi-directional pedestrian flow usually forms two contraflows by stratification. As pedestrian density increases, the two contraflows will impede each other, resulting in jamming and clogging.

Currently, there are many simulation research models on pedestrian flow, which can be divided into macroscopic and microscopic aspects. Macro models focus on the formation mechanisms of macroscopic pedestrian flow characteristics and phenomena in different environments from a global perspective. Micro models take individual pedestrians as the research object to describe individual movement in various environments. Recently, simulation research exploring macroscopic pedestrian flow movement mechanisms and characteristics in different environments, based on studying microscopic pedestrian characteristics, has become a focus in pedestrian flow research. Among these, the cellular automata (CA) model is the most widely used simulation model. CA can be used to study macroscopic behavioral characteristics by simulating microscopic pedestrian behaviors in the system. There is considerable literature on bi-directional pedestrian flow based on the CA model.

Blue and Adler^1–3^ defined two simple movement rules: lane changing and forward movement, and presented the use of CA micro-simulation for modeling bi-directional pedestrian walkways. They showed that there are three modes of bi-directional pedestrian flow: (a) interspersed flow, (b) flows in directional separated lanes, and (c) dynamic multi-lane flow. Based on Blue's work, Fang et al.^4^ proposed a CA model assuming pedestrians can only move in four directions. They revealed that most pedestrian 'back-step strategies' can significantly reduce jamming conditions. By observing the behavior of several pedestrians in a scene, Wu and Guo^5^ proposed that pedestrian movement is driven by a potential field, and established a microscopic pedestrian cellular automata model in discrete space to simulate pedestrian flow through multiple queues, obtaining ways to help improve pedestrian mobility. The essence of these CA models is to study the transition probability of pedestrian movement. A pedestrian's transition probability not only embodies the characteristics of pedestrian movement but also plays an important role in the emergence of self-organization phenomena in pedestrian flow. Consequently, the transition probability is a key parameter when using CA models to simulate bi-directional pedestrian flow.

Kretz et al.^6^ conducted counter-current experiments on bi-directional pedestrian flow in a straight corridor, deeply studying pedestrian evacuation time, pedestrian speed, system volume, stratification formation, symmetry destruction, etc. Moussaïd et al.^7^ found that the difference in pedestrian speeds is a key factor for instability in pedestrian flow stratification. When all pedestrians walk at the group's average speed, the evacuation efficiency of the bi-directional pedestrian flow reaches the highest level. However, in reality, due to local interactions between slow and fast pedestrians, the stratification is destroyed, reducing pedestrian movement efficiency. Feliciani and Nishinari^8^ experimentally studied the formation mechanism of the stratification phenomenon during bi-directional pedestrian flow. The results show the most stable stratification forms with balanced bi-directional flows, but compared to unbalanced flows, balanced flow requires pedestrians to have more space for lateral movement. At low densities, balanced bi-directional flow has the highest evacuation efficiency, but at high densities, jamming and deadlock clogging can occur. Xu et al.^9^ quantitatively described bi-directional pedestrian flow scenarios by introducing a minimum expected collision-free velocity model into pedestrian flow dynamics. The simulations show the minimum expected collision-free velocity effectively accelerates bi-directional flow stratification and reduces congestion. Therefore, studying the stratification mechanism of bi-directional pedestrian flow is key to improving pedestrian evacuation efficiency.

In scenes of bi-directional pedestrian flow, increasing numbers of researchers are studying pedestrian movement characteristics by setting up specific facilities or introducing specific movement rules, exploring key factors affecting pedestrian flow efficiency, and providing support for formulating pedestrian control strategies. Helbing et al.^10^ analyzed two opposing pedestrian flows passing through a narrow gate, finding the flows oscillate alternately, with each direction passing through separately over time. Yang et al.^11,12^ discussed right-moving preference effects on pedestrian movement by simulating bi-directional flow in a corridor. They found that right-moving preference is very effective for evacuating high-density bi-directional flow. Guo et al.^13^ and Jin et al.^14^ simulated bi-directional movement considering restricted pedestrian sightlines and sideways behavior respectively, further expanding bi-directional flow research scope. Molyneaux et al.^15^ proposed a dynamic traffic control framework for bi-directional flow by simulating gated and gateless scenarios and controlling gate usage time in gated cases. Jin et al.^16^ studied bi-directional flow characteristics under different corridor widths, obtaining the effect of width on average pedestrian velocity.

Over several decades, some researchers have conducted actual evacuation experiments to obtain key parameters for CA models, making pedestrian flow simulations more realistic. For instance, Shields and Boyce^17^ carried out unannounced drills in four retail stores, collecting data through experiments and questionnaires to verify evacuation models. Yang et al.^12^ conducted large-scale evacuation experiments in a university building channel using typical student groups, using measured data as CA model parameters to simulate pedestrian movement characteristics. Zhang et al.^18^ proposed a synthetic approach to recognize and identify large pedestrian flows. Liberto et al.^19^ calibrated the model parameters with a behavioral-based approach that relies on observed movement behaviors. Nishihara^20^ revealed two interaction force fields in pedestrian movement: personal space as a repulsive force, and information processing space as an attractive force. During movement, pedestrians imitate others while maintaining distance. Therefore, the repulsive force of personal space and the attractive force of information space both influence pedestrians' next-step positions.

However, few studies have examined how to determine personal space and information processing space. Most studies uniformly define these spaces based on statistical experience, without accounting for differences in pedestrians' spatial judgment. Therefore, studying this difference is an important concern. Guo et al.^21^ found through controlled experiments that in the bi-directional flow, each pedestrian's speed relates not only to surrounding density but also to same-direction density ahead. Pedestrians within 1 m ahead in the same direction have the greatest impact on speed. Li and Niu^22^, Li et al.^23,24^ found heterogeneity in pedestrians' judgment of interaction force fields. This heterogeneity introduces obvious uncertainty and dynamism into personal decision-making and movement methods.

In summary, research on bi-directional pedestrian flow simulation based on CA models demonstrates macro-level complex phenomena emerging from micro-level individual interactions and finally reveals the evolution mechanism and macroscopic behavior of this complex system.

Based on some limitations of existing bi-directional pedestrian flow models, this paper proposes a direction fuzzy visual field (DFVF) to describe the randomness of pedestrian movement within personal space and information processing space, as well as express the difference in pedestrian behavior. It applies various static and dynamic factors in the DFVF to simulate bi-directional pedestrian movement in a corridor, analyzing the effects of pedestrian density and system scale on evacuation time. It also illustrates pedestrian flow density-speed and density-volume curves, observes self-organization phenomena during simulation, and presents pedestrian interactions to reveal inherent laws of bi-directional pedestrian flow in the complex system.

## Direction fuzzy visual field

Even within the same environment, pedestrians in facilities exhibit heterogeneity in psychological traits like cognition, emotion, attitude, and needs. This results in obvious differences when they determine the range of interaction force fields. Therefore, the traditional CA model's assumption of no differences in how pedestrians determine the interaction force field range is inappropriate.

To study differences in how pedestrians judge the range of interaction force fields, we designed a controlled experiment to determine range sizes. By examining heterogeneity across different pedestrians experimentally, we can establish a model capable of more finely simulating the movement characteristics of pedestrian flow.

### Problem definition and formulation

**Figure 1:**
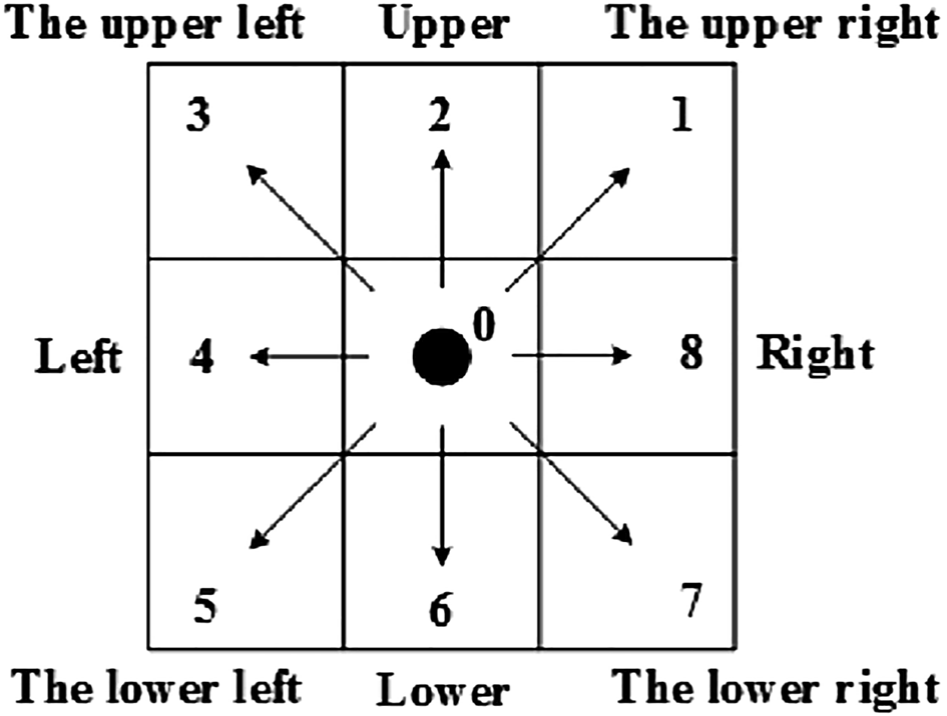
The possible direction of pedestrian movement in the Moore neighborhood. There are 8 directions for pedestrians to choose from in the Moore neighborhood.

In the CA model, pedestrians can only move towards their neighbors. In addition to holding position, pedestrians will have a movement direction when they move to other positions in the neighborhood. There are 8 directions for pedestrians to choose from the Moore neighborhood, as shown in Fig. 1.

A key problem in model construction is how pedestrians determine the choice of neighbors in different directions when interacting with static and dynamic information in the range of the interaction force field. Pedestrians divide the whole force field into areas in different directions according to their characteristics (e.g. Moore neighborhood has 8 directional areas). Then, pedestrians can decide to move towards the neighbor in a certain direction according to the interaction with static and dynamic information in different directional areas. Here, we define different directional areas as Directional Visual Fields (DVF). DVF is an important factor affecting how pedestrians move.

**Figure 2:**
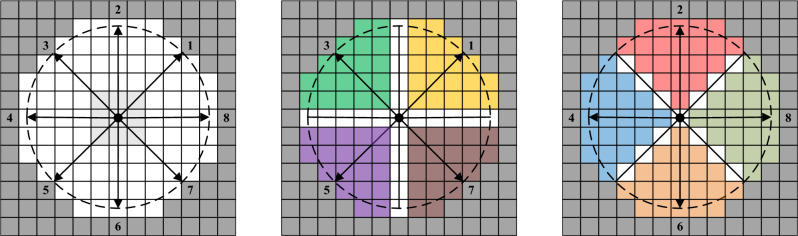
The DVF is based on a circle with a radius of 5 cell lengths.

In the process of movement, pedestrians will obtain static and dynamic information through their senses. When turning around is considered, pedestrians' visual and auditory field is approximately a circle. Therefore, it is common to assume that the overall interaction force field of pedestrians is the circle. When the DVF is divided in the circular area, it is divided equally in 8 directions according to the characteristics of the Moore neighborhood. Figure 2 shows the DVF based on the circle with a radius of 5 cell lengths in the condition of a Moore neighborhood. The pedestrian's interaction force field is divided into equal areas in 8 directions, and there is considerable overlap between the adjacent DVFs.

In essence, the DVFs based on the circle equally divide the interaction force field into 8 areas according to the Moore neighborhood, and each pedestrian has the same size of DVF. However, the obvious heterogeneity of pedestrians' psychological characteristics affects their judgment of the DVF range. Therefore, when different pedestrians judge DVF, it is not assumed that there is no difference in most CA models. To show the differences of pedestrians in the DVF selection through control experiments is the key to refining and improving the CA model.

### Building experiments scene

Even in the same environment, different pedestrians in pedestrian facilities have obvious heterogeneity in the judgment of DVF due to the differences in psychological characteristics such as cognition, emotion, attitude, and demand. To study the difference in pedestrians' DVF, the experimental scene in the judgment of DVF's range is designed by using the method of control experiment. Through the experiment, the heterogeneity of pedestrians' DVF is studied, to make the established model more refined and efficiently simulate the movement characteristics of pedestrian flow.

**Figure 3:**
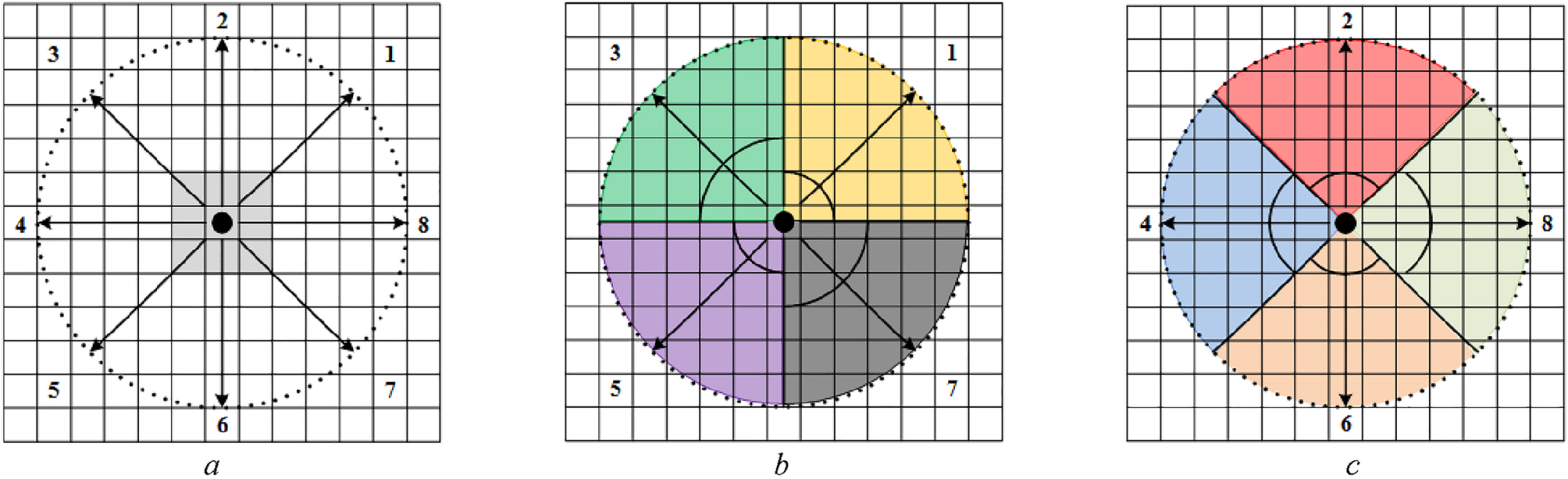
Pedestrians' overall interaction force field and DVF. (**a**) The overall interaction force field, (**b**) The maximum DVF on direction 1,3,5,7, (**c**) The maximum DVF on direction 2,4,6,8

To reflect the behavior of pedestrians such as moving forward, changing lanes, waiting, and stepping back, the Moore neighborhood is used in the control experiment, as shown in Fig. 1. Assuming that pedestrians have no special requirements for the range shape when judging DVF, the overall interaction force field is defined as a circle with a radius of *r*. Figure 3 depicts the overall interaction force field with a radius *r* of 5 cell lengths, from which it can be seen that the maximum angle of DVF in each direction is 90°.

**Figure 4:**
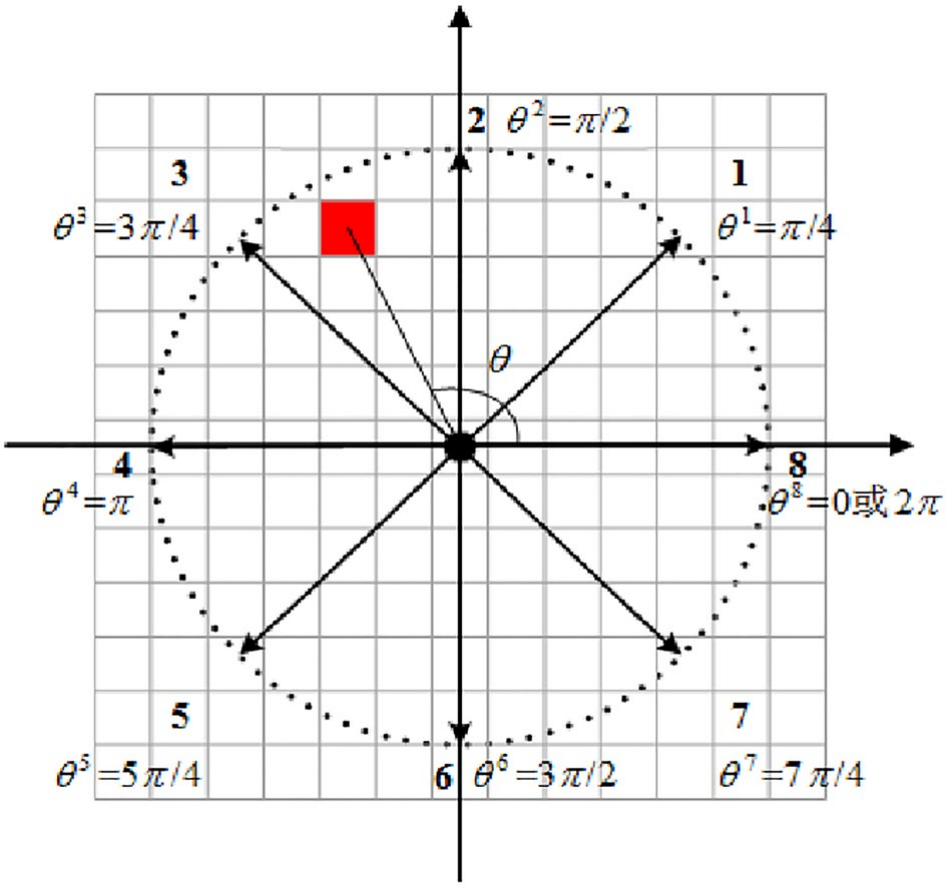
Schematic diagram of angle θ in pedestrians' DVF coordinate system.

To describe the pedestrians' DVF, the control experiment specifies that the pedestrian is used as the coordinate origin to construct a two-dimensional coordinate system. In the pedestrian interaction force field, the intersection angle between each cell center and the coordinate origin is marked as *θ*, as shown in Fig. 4, where a black circle expresses a pedestrian, and a red square indicates a cell as a study object. Therefore, as can be seen from Fig. 4, direction 1 has angle *θ*^1^ = *π*/4, direction 2 has angle *θ*^2^ = *π*/2, direction 3 has angle *θ*^3^ = 3*π*/4, direction 4 has angle *θ*^4^ = *π*, direction 5 has angle *θ*^5^ = 5*π*/4, direction 6 has angle *θ*^6^ = 3*π*/2, direction 7 has angle *θ*^7^ = *π*/4, and direction 8 has angle *θ*^8^ = 0 or *θ*^8^ = 2*π*.

When a pedestrian decides whether a cell belongs to this DVF, his or her decision is based on the absolute value $x$ of the difference between the cellular intersection angle $\theta$ and the direction’s angle $\theta^{d}$($d = 1,2, \cdots ,8$), shown as formula (1). If the absolute value $x$ of the difference between $\theta$ and $\theta^{d}$ is within the range of $[0,{\pi \mathord{\left/ {\vphantom {\pi 4}} \right. \kern-0pt} 4}]$, it is determined that the cell is within the DVF range of direction $d$.

1 $$x = \left\{ {\begin{array}{*{20}l} {\left| {\theta - \theta^{d} } \right|,} & {d = 1,2, \cdots ,7} \\ {\min \left\{ {\left| {\theta - 0} \right|,\left| {\theta - 2\pi } \right|} \right\},} & {d = 8} \\ \end{array} } \right.$$

**Figure 5:**
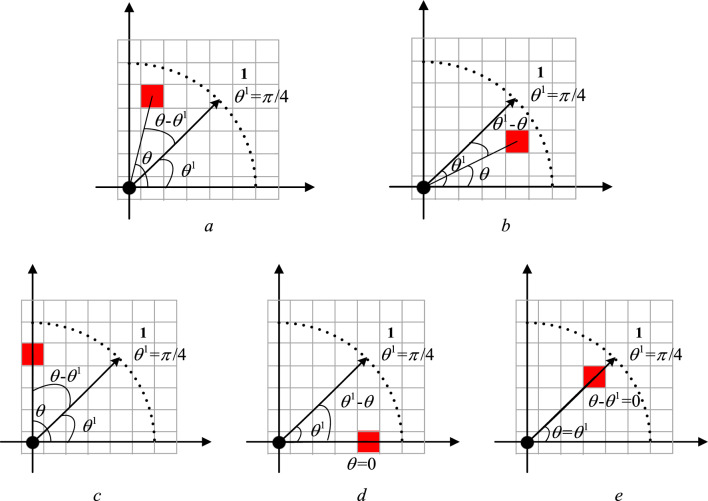
Calculation diagram of x value when d = 1 (r = 5). (**a**) $\theta > \theta^{{1}}$, (**b**) $\theta < \theta^{{1}}$ (**c**) $\theta { = }\pi {/2}$, (**d**) $\theta { = 0}$, (**e**) $\theta { = }\theta^{{1}}$.

Figure 5 shows the calculation process of $x$ when $d = 1$ in the condition of $r = 5$.

**Figure 6:**
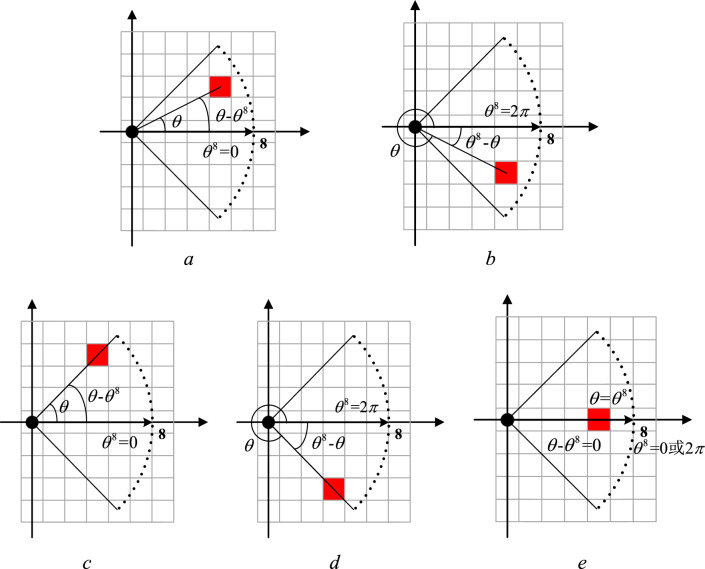
Calculation diagram of x value when d = 8 (r = 5). (**a**) $\theta \in (0,\pi {/4}) \to \theta^{{8}} = 0$, (**b**) $\theta \in (7\pi {/4},2\pi ) \to \theta^{{8}} = 2\pi$, (**c**) $\theta { = }\pi {/4} \to \theta^{{8}} = 0$, (**d**) $\theta { = 7}\pi {/4} \to \theta^{{8}} = 2\pi$, (**e**) $\theta { = }\theta^{{8}}$.

Figure 6 demonstrates the calculation process of $x$ when $d = 8$.

**Figure 7:**
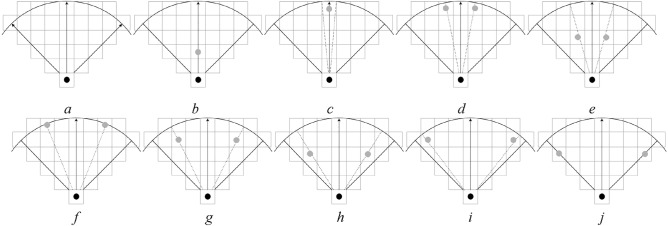
Test scenarios with DVF radius $r = 5$. (**a**) The maximum valid range in a certain direction, (**b**) $x = 0$, (**c**) $x = \pi {/32}$, (**d**) $x = \pi {/16}$, (**e**) $x = {3}\pi {/32}$, (**f**) $x = \pi {/8}$, (**g**) $x = {5}\pi {/32}$, (**h**) $x = {3}\pi {/16}$, (**i**) $x = {7}\pi {/32}$, (**j**) $x = \pi {/4}$.

**Figure 8:**
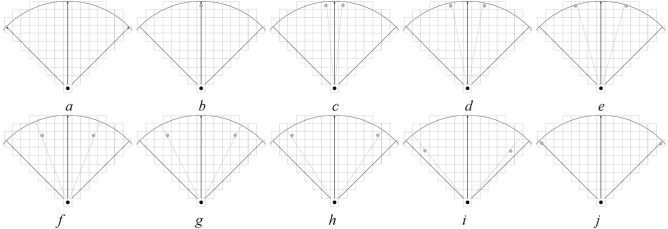
Test scenarios with DVF radius $r = 10$. (**a**) The maximum valid range in a certain direction, (**b**) $x = 0$, (**c**) $x = \pi {/32}$, (**d**) $x = \pi {/16}$, (**e**) $x = {3}\pi {/32}$, (**f**) $x = \pi {/8}$, (**g**) $x = {5}\pi {/32}$, (**h**) $x = {3}\pi {/16}$, (**i**) $x = {7}\pi {/32}$, (**j**) $x = \pi {/4}$.

**Figure 9:**
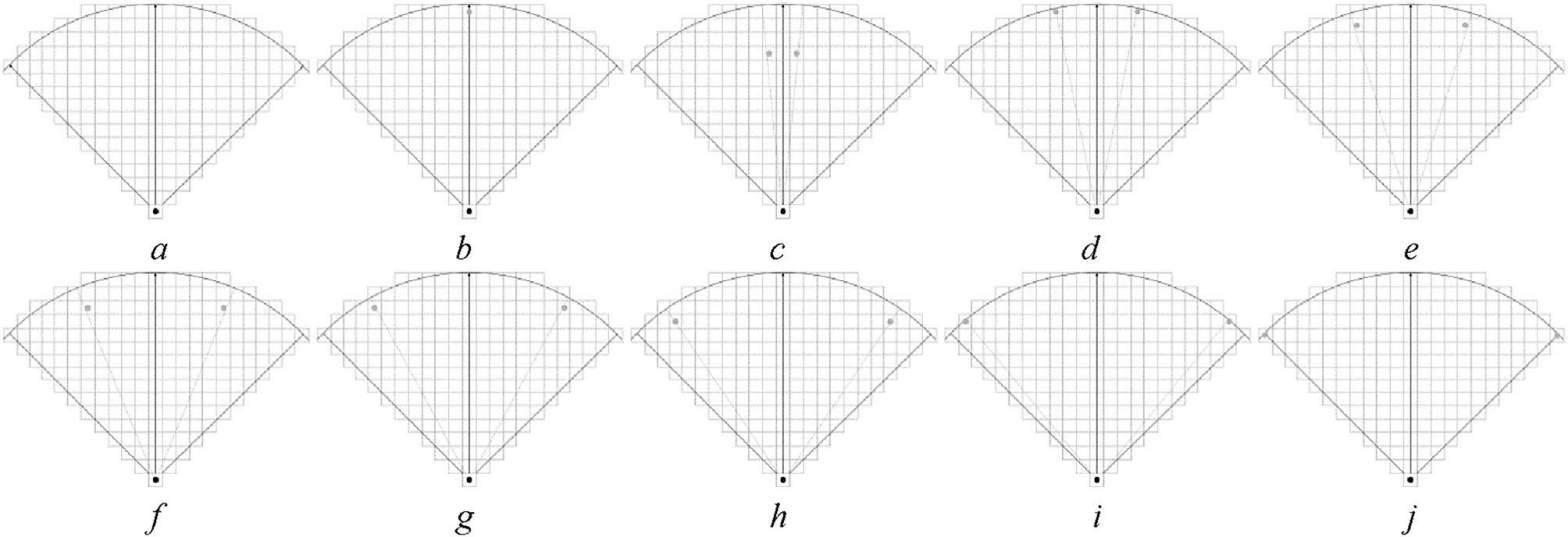
Test scenarios with DVF radius $r = 15$. (**a**) The maximum valid range in a certain direction, (**b**) $x = 0$, (**c**) $x = \pi {/32}$, (**d**) $x = \pi {/16}$, (**e**) $x = {3}\pi {/32}$, (**f**) $x = \pi {/8}$, (**g**) $x = {5}\pi {/32}$, (**h**) $x = {3}\pi {/16}$, (**i**) $x = {7}\pi {/32}$, (**j**) $x = \pi {/4}$.

To study the difference in pedestrian DVF, a DVF judgment experimental scene with interaction force field radius $r$ of 5, 10, and 15 cell lengths is designed by using the method of control experiment. To simplify the control experiment, nine values with equal spacing (0, $\pi {/32}$, $\pi {/16}$, ${3}\pi {/32}$, $\pi {/8}$, ${5}\pi {/32}$, ${3}\pi {/16}$, ${7}\pi {/32}$, $\pi {/4}$) were chosen in $x \in [0,\pi {/4]}$, and test scenarios with radiuses $r = 5,10,15$ cells were constructed. Figures 7, 8, and 9 show the test scenarios with the radius $r = 5,10,15$ cells. In the test scenario, the organizer (grey circle) stood in a predefined position and asked the participant (black circle) to judge whether the pedestrian at the predefined position was in the DVF.

### Data analysis

Following the experimental design with control groups established by Sun and Elefteriadou^25^, we implemented a control experiment on pedestrians' DVF judgment in major universities, railway stations, and bus stations in Lanzhou, Gansu Province, China (see, e.g.,^22–24^). Data were collected by questionnaire. First, because of the difference in pedestrians' cultural level and the low participation degree of pedestrians, the proportion of college students in the questionnaires is large (65.3%). Second, pedestrian drop-out rates are relatively high due to various reasons, especially at the station. Therefore, it is difficult to deal with the validity of the questionnaires in the later stage. We classified the questionnaires and combined some incomplete questionnaires (22.8%) with the Variance Homogeneity Test, Factor Analysis Approach, and Significance Test of Difference. Finally, this control experiment collected a total of 1893 valid questionnaires. The questionnaire frequency statistics are listed in Table 1.

**Table 1:** Questionnaire frequency statistical results (unit: %).

$r$	Defined scenario $x$								
	0	$\pi {/32}$	$\pi {/16}$	${3}\pi {/32}$	$\pi {/8}$	${5}\pi {/32}$	${3}\pi {/16}$	${7}\pi {/32}$	$\pi {/4}$
$r{ = 5}$	100	99.3	82.6	72.8	55.9	38.6	11.4	3.1	0.3
$r{ = 10}$	99.4	92.8	80.7	70.3	54.6	37.8	16.2	4.3	1.1
$r{ = 15}$	99.8	90.9	84.8	60.7	53.3	34.5	16.1	5.1	0.9
Average	99.7	94.3	82.7	67.9	54.6	37.0	14.6	4.2	0.7

The study was performed according to the Declaration of Helsinki. The experimental protocols were approved by the Gansu Provincial Department of Transportation, the Gansu Provincial Department of Science and Technology, and the Academic Ethic Committee of Lanzhou Jiaotong University. Informed consent was obtained from all participants before they took part in the survey. This study obtains, stores, manages, interprets, analyzes, and applies data in a manner consistent with ethical standards and social responsibility.

The questionnaire results indicate that pedestrian's judgment of DVF is a fuzzy process, named direction fuzzy visual field (DFVF). Therefore, the fuzzy membership function $A(x)$ of DFVF is key to addressing this problem.

The method of assigning a membership function is used to measure the DFVF of pedestrians. The method of assigning membership function is generally considered to take people's practical experience into account, apply some existing fuzzy distribution according to the nature of the problem, and then determine the parameters of the fuzzy distribution based on the measured data.

**Figure 10:**
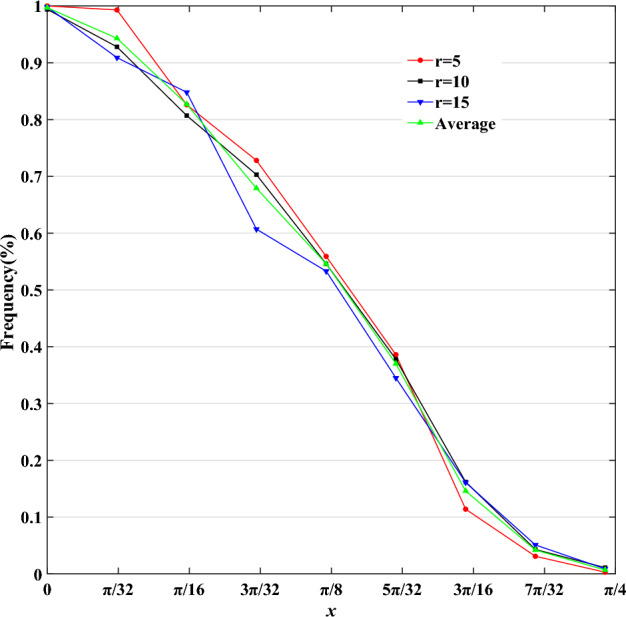
Trend chart of frequency.

Therefore, according to the judgment characteristics shown in Table 1, Fig. 10 shows the trend of frequency change with $x$. It can be found that this tendency approximately follows a descending half-mountain-shaped distribution, denoted as formula (2).

2 $$A(x) = \left\{ {\begin{array}{*{20}l} {1,} \hfill & {x = 0} \hfill \\ {{0}{\text{.5}} - 0.5\sin a(x - b),} \hfill & {0 < x \le \pi {/4}} \hfill \\ {0,} \hfill & {x > \pi /4} \hfill \\ \end{array} } \right.$$

where $a$ and $b$ are parameters of descending half-mountain-shaped distribution.

**Figure 11:**
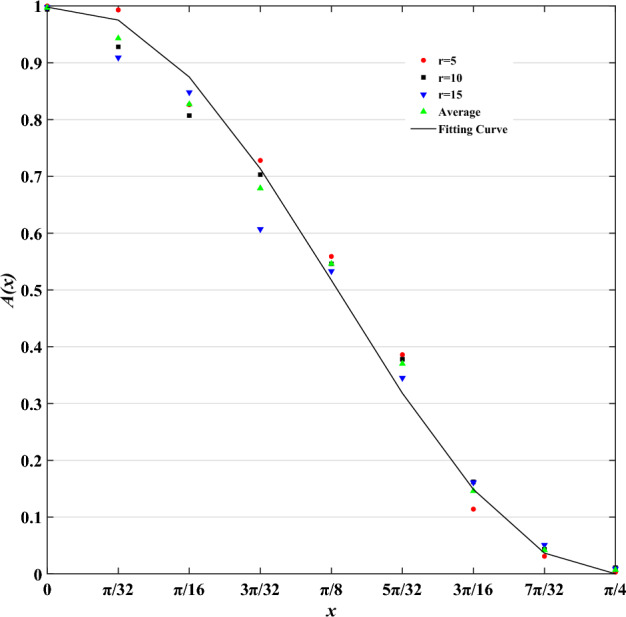
An illustration of a fitting curve.

We fitted the data in Table 1 with Formula (2). The fitting results are shown in Formula (3) and Fig. 11.

3 $$A^{d} (x) = \left\{ {\begin{array}{*{20}l} {1,} \hfill & {x = 0} \hfill \\ {{0}{\text{.5}} - 0.5\sin 4.14{2}(x - {0}{\text{.401}}),} \hfill & {{\kern 1pt} 0 < x \le \pi {/4}} \hfill \\ {0,} \hfill & {x > \pi /4} \hfill \\ \end{array} } \right.$$

where $d = 1,2, \cdots ,8$ and $A^{d} (x)$ denotes the fuzzy distribution of the DFVF in direction $d$.

**Figure 12:**
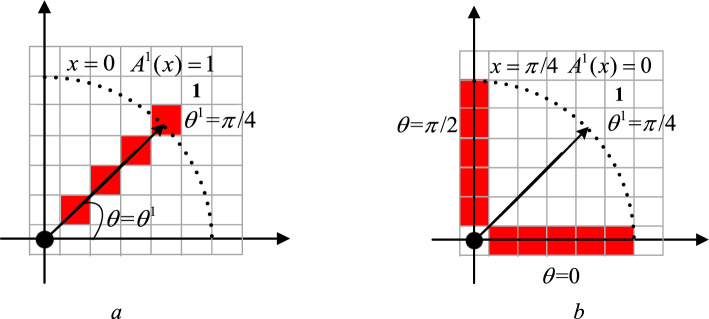
Schematic diagram of fuzzy probability of red cell belonging to direction 1 (r = 5). (**a**) $x = 0$, (**b**) $x = \pi {/4}$.

As shown in Fig. 12, when $x = 0$, the cells on $x$ are just in direction $d$, then the fuzzy membership function $A^{d} (x)$ determines that these cells belong to direction $d$, and the fuzzy probability of DFVF is 1; when $x = \pi {/8}$, the range within the included angle of $x$ is exactly half of the maximum feasible range of DVF, then $A^{d} (x)$ determines the fuzzy probability that the cell on $x$ belongs to the DFVF of $d$ is 0.5; when $x = \pi {/4}$, the cells on $x$ are just in the adjacent directions of direction $d$ at this time, and the fuzzy probability of $A^{d} (x)$ determining that the cell on $x$ belongs to the DFVF of $d$ is 0.

To describe the differences of pedestrians in the DFVF, we assume that the fuzzy distribution $A^{d} (x)$ in formula (3) has the following $\forall \;\lambda \in [0,1][0,1]$:

4 $$A_{\lambda }^{d} (x)\mathop {\overline{\overline{{{\kern 1pt} {\kern 1pt} {\kern 1pt} {\kern 1pt} {\kern 1pt} {\kern 1pt} {\kern 1pt} {\kern 1pt} {\kern 1pt} {\kern 1pt} {\kern 1pt} {\kern 1pt} {\kern 1pt} {\kern 1pt} {\kern 1pt} {\kern 1pt} {\kern 1pt} {\kern 1pt} {\kern 1pt} {\kern 1pt} {\kern 1pt} {\kern 1pt} {\kern 1pt} {\kern 1pt} {\kern 1pt} {\kern 1pt} {\kern 1pt} }}} }\limits^{{{\text{def}}}} \{ x|A^{d} (x) \ge \lambda \}$$

$A_{\lambda }^{d} (x)$ is the $\lambda$-cut set of $A^{d} (x)$, where $\lambda$ is the threshold or confidence level.

Formula (4) shows that $\lambda$-cut set $A_{\lambda }^{d} (x)$ is a classic set, composed of members with a degree of membership equal to or above $\lambda$. Its characteristic function is:

5 $$\chi_{{A_{\lambda }^{d} (x)}} = \left\{ {\begin{array}{*{20}l} {1,} & {A^{d} (x) \ge \lambda } \\ {0,} & {A^{d} (x) < \lambda } \\ \end{array} } \right.$$

To simplify the simulation process, we assume that the choice of the DFVF on different pedestrians is completely random in the process of simulation. When any pedestrian is deciding upon his DFVF, a random number in [0, 1] is selected for $\lambda$ to be the threshold for this pedestrian in $A^{d} (x)$. Subsequently, the characteristic function $A_{\lambda }^{d} (x)$ is used in the decision.

Pedestrians' DFVF can effectively describe the heterogeneity of pedestrian interaction force field selection and can explain the phenomenon that there is no regional overlap or partial regional overlap between adjacent DFVFs through a fuzzy concept. This phenomenon cannot be represented by the traditional field models. When a pedestrian determines the DFVF, if there is no overlapping region between its adjacent DFVF, it indicates that the pedestrian has a narrow DVF, the adjacent DVFs have clear boundaries, and the pedestrian pays more attention to the maintenance of direction. If there are overlapping areas, it means that the pedestrian has a wide DVF, and the adjacent areas have no clear boundaries. The determination of DVF is a fuzzy concept. If the overlapping areas are larger, the definition of DVF is fuzzier, and the pedestrian is more inclined to constantly adjust the direction to move to the target position. This DFVF determination aligns with current observations of real-world pedestrian flows.

## Model

**Figure 13:**
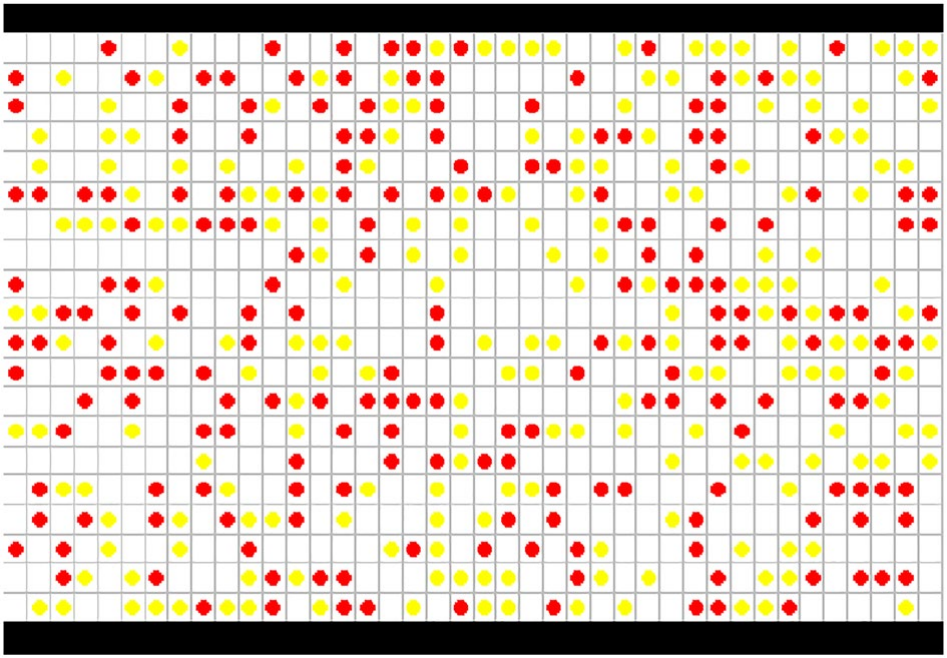
The model of bi-directional pedestrian flow based on CA.

The model is described in the $L \times W$ two-dimensional cellular system $\Omega^{2}$, where $L$ is the length, $W$ is the width, and $\Omega^{2}$ is the cellular system. The corridor exits are located on the left and right sides of the cellular system. The size of a cell corresponds to approximately 0.4 m × 0.4 m (see, e.g.^4,11^. Based on empirical statistics, the average speed of a pedestrian is about 1.00 m/s (see, e.g.^4,11^). Therefore, a time-step is 0.4 s in the model. The pedestrian flow movement is divided into two directions based on the left and right proportion of pedestrians. There are two ways to process when a pedestrian moves to an exit: (a) this pedestrian will leave the system, (b) if this pedestrian is at the left exit, he or she will enter from the right exit, and if this pedestrian is at the right exit, he or she will enter from the left exit. This model is demonstrated in Fig. 13, where 'red circle' shows a pedestrian moving leftward, and 'yellow circle' indicates a pedestrian moving rightward. The initial states of pedestrians are randomly distributed throughout the system based on prescribed proportions of left-moving and right-moving pedestrians.

**Figure 14:**
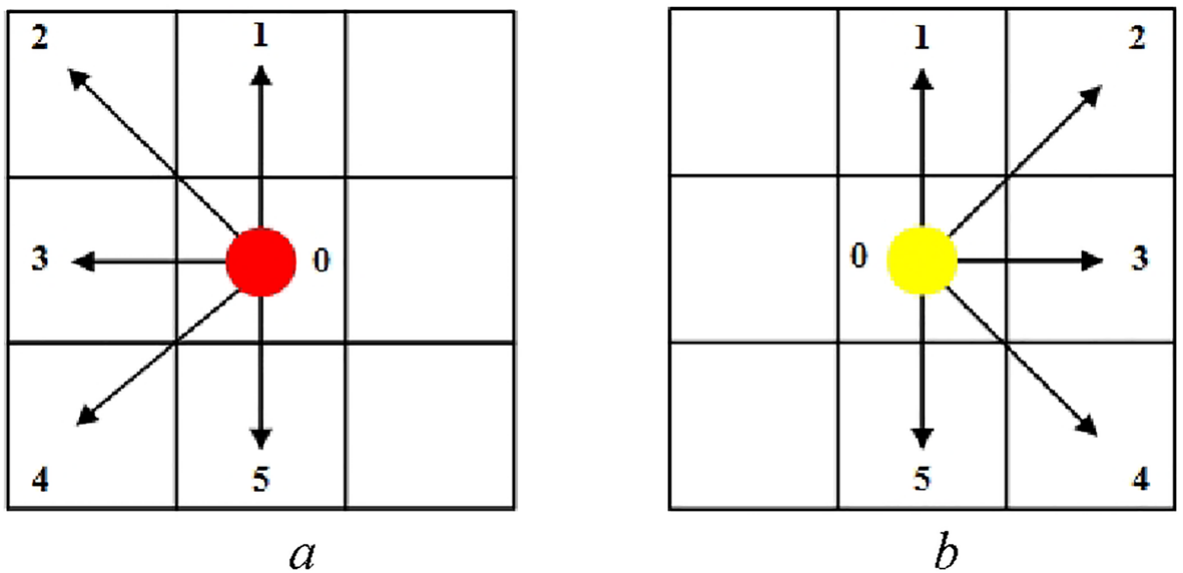
The target positions of pedestrian movement. (**a**) A pedestrian moves leftward, (**b**) a pedestrian moves rightward.

The model assumes that pedestrians can only move forward, change lanes, or wait in the situ, there is no backward behavior. Therefore, every pedestrian has 6 target positions to choose from based on the Moore neighborhood, as shown in Fig. 14.

### Fuzzy space

**Figure 15:**
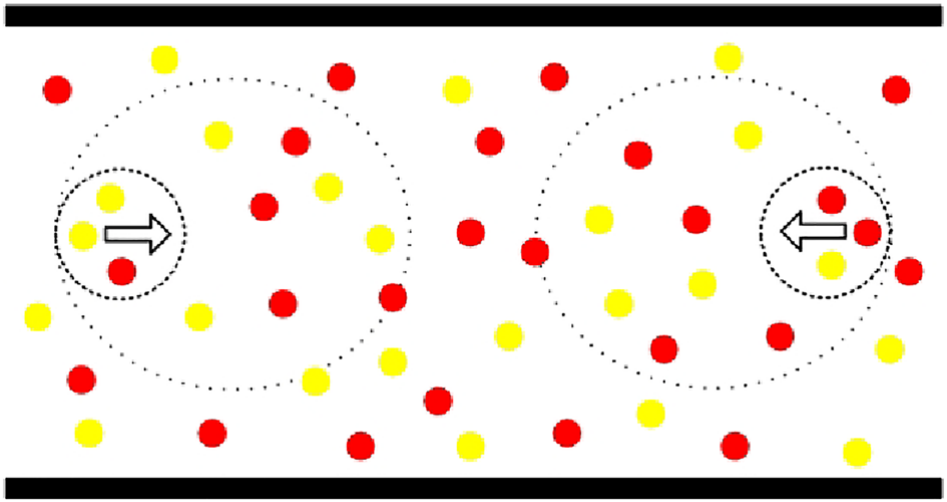
The personal space and information processing space.

During movement, pedestrians will not only follow others but also try to keep a distance from others. This means that there are two dynamic action fields: (a) a repulsive force field named personal space, and (b) a gravitational force field defined as information processing space^20^. Pedestrians unconsciously divide their territory into personal space. Once someone gets too close to another, it will make most feel uncomfortable. Therefore, the pedestrian personal space is a repulsive force field in the process of movement. Apart from that, pedestrians have a certain herd mentality and will follow others in a certain range. This range is named pedestrian information processing space^20^. The pedestrian information processing space is a gravitational force field, which attracts pedestrians to maintain a certain flow direction. A pedestrian personal space and information processing space are shown in Fig. 15, where a small circle stands for a pedestrian personal space, and a big circle represents a pedestrian information processing space. Based on empirical statistics, the radius of personal space is approximately 2 m; the radius of information processing space is about 5 m^20^.

During movement, the personal space and information processing space jointly affect the choice of every pedestrian target position. When calibrating a pedestrian personal space and information processing space in the CA model, we suppose that these two force fields are composed of cells. Assuming that the two-dimensional cellular system has no boundaries, and each pedestrian's force field has enough cells. Therefore, a pedestrian personal space is a region that is enlarged to 2 m based on his or her neighborhood (as shown in Fig. 14), and a pedestrian information processing space is an area that is enlarged to 5 m. In a pedestrian personal space, if a pedestrian is near the system boundary, the model supposes that there are in-existent cells that are all occupied by opposite pedestrians, this hypothesis shows that pedestrians do not like to crowd near the wall, which has a repulsive force on pedestrians. In a pedestrian information processing space, if a pedestrian is near the wall, the model assumes that there are in-existent cells that are all occupied by pedestrians in the same direction. Contrary to personal space, this hypothesis demonstrates that the wall has a gravitational force on pedestrians; the wall can help pedestrians effectively reach the exit. Thus, it can be seen that the wall also has two opposite forces on pedestrians.

**Figure 16:**
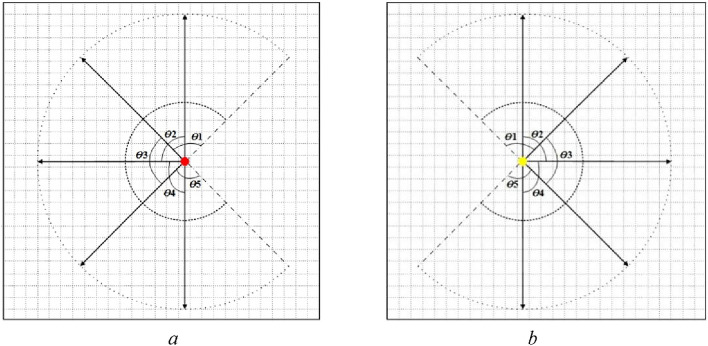
The maximum range of personal space and information processing space in CA. (**a**) A pedestrian moves leftward, (**b**) a pedestrian moves rightward.

In empirical statistics, pedestrians can still see the left and right sides in the forward process of movement. The maximum angle of DVF in each direction is 90° when a pedestrian judges his or her personal space and information processing space in a certain direction as shown in Fig. 14. Consequently, the maximum angle vision of a pedestrian is 270° in the process of movement. Figure 16 shows the maximum range of personal space and information processing space in the CA model, where $\theta_{{1}} = \theta_{{2}} = \theta_{{3}} = \theta_{{4}} = \theta_{{5}} = {90}^{ \circ }$.

To show the heterogeneity of different pedestrians in a force field selection, based on the definition of direction fuzzy visual field, DFVFs of a pedestrian $p$ in personal space ($r = 5$, the radius is 2 m) and information processing space ($r = 12.5$, the radius is 5 m) in five directions are constructed. This method can finely describe the heterogeneity of pedestrians in force field selection.

### Direction force parameter

A pedestrian does not interact with all neighbors in the process of movement. Ballerini et al.^26^ revealed that the interaction does not depend on the metric distance, as most current models and theories assume, but rather on the topological distance through research on interaction ruling animal collective behavior. They discovered that each member interacts on average with a fixed number of neighbors (six or seven), rather than with all neighbors within a fixed metric distance.

To simulate pedestrian interaction in a simplified manner, empirical data from Ballerini et al.^26^ was directly adopted, and the interaction was depicted based on the principle of $k$-nearest neighbors ($k = {6}$). When the number of cells associated with a pedestrian is not less than six, we choose the nearest six cells to interact with this pedestrian in his or her personal space or information processing space. If the number is not more than six, we select all cells to interact with him or her.

For bi-directional pedestrian flow, a pedestrian should move to a target position in his or her personal space, which has more empty cells to keep a certain distance from others in the next time-step. Hence, this paper assumes that $\Omega^{2}$ is a two-dimensional discrete cells system, $\dot{S}_{d}^{p} \subset \Omega^{2}$ ($d = 0,1, \cdots ,5$) is expressed as the personal space on direction $d$ for a pedestrian $p$. It chooses the nearest six empty cells as the action scope in $\dot{S}_{d}^{p}$. If the number of empty cells associated with $p$ is not more than six, it uses the cell of $p$ to supplement every insufficient cell. $d^{\prime}_{k}$ ($k = 1,2, \cdots ,6$) is described as the Euclidean distance from $p$ to a cell in the interaction force field. Then we have that

6 $$\dot{E}_{d}^{p} = \frac{1}{6(6 - 1)}\sum\limits_{i = 1}^{6} {\frac{1}{{d_{k}^{\prime } }}}$$

is called the efficiency of $\dot{S}_{d}^{p}$ for $p$, and $\dot{E}_{0}^{p}$ is set zero.

Equation (6) shows that the efficiency of personal space is a mean value of $d^{\prime}_{k}$ reciprocal sum, which is expressed as the average reachability of the personal space on direction $d$.

In the information processing space, a pedestrian will be attracted by other pedestrians in the same direction, and he or she should move to a target position in his or her information processing space, which has more cells occupied by other pedestrians in the same direction in the next time-step. Hence, we suppose that $\ddot{S}_{d}^{p} \subset \Omega^{2}$ ($d = 0,1, \cdots ,5$) is expressed as the information processing space in the direction $d$ for a pedestrian $p$ in $\Omega^{2}$. And we select the nearest six cells occupied by other pedestrians in the same direction as the action scope in $\ddot{S}_{d}^{p}$. If the number of cells associated with $p$ is not more than six, we use the cell of $p$ to supplement every insufficient cell. $d^{\prime\prime}_{k}$($k = 1,2, \cdots ,6$) is described as the Euclidean distance from $p$ to a cell in the interaction force field. Then we have that

7 $$\ddot{E}_{d}^{p} = \frac{1}{6(6 - 1)}\sum\limits_{i = 1}^{6} {\frac{1}{{d_{k}^{\prime \prime } }}}$$

is called the efficiency of $\ddot{S}_{d}^{p}$ for $p$, and $\ddot{E}_{0}^{p}$ is set zero.

Equation (7) shows that the efficiency of information processing space is a mean value of $d^{\prime\prime}_{k}$ reciprocal sum, which is expressed as the average reachability of the information processing space on direction $d$.

To synthesize a repulsive force of personal space and a gravitational force of information processing space, this paper assumes that $lp$ is a pedestrian moving leftwards in $\Omega^{2}$, $(i,j)$ is his or her current position's coordinate, his or her 6 optional position's coordinate is $(x,y) \in \{ i - 1,i\} \times \{ j - 1,j,j + 1\}$. Then we have that

8 $$F_{d}^{lp} = \left\{ {\begin{array}{*{20}l} {0,{\kern 1pt} } & {(x,y) = (i,j)} \\ {\omega {\kern 1pt} {\kern 1pt} {\kern 1pt} \dot{E}_{d}^{lp} + (1 - \omega ){\kern 1pt} {\kern 1pt} \ddot{E}_{d}^{lp} ,} & {(x,y) \ne (i,j)} \\ \end{array} } \right.$$

is called the direction force parameter of $$lp$$ on direction $$d$$. Thereinto, $$\{ i - 1,i\} \times \{ j - 1,j,j + 1\}$$ is shown as the set of Cartesian products between $$\{ i - 1,i\}$$ and $$\{ j - 1,j,j + 1\}$$, $$\omega$$$$(0 \le \omega \le 1)$$ is the inertia weight, $$\omega$$ is taken as 0.5 in this paper.

Again, if $rp$ is a pedestrian moving rightwards, $(i,j)$ is his or her current position's coordinate, his or her 6 optional position's coordinate is $(x,y) \in \{ i,i + 1\} \times \{ j - 1,j,j + 1\}$. Then we have that

9 $$F_{d}^{rp} = \left\{ {\begin{array}{*{20}l} {0,} & {(x,y) = (i,j)} \\ {\omega {\kern 1pt} {\kern 1pt} {\kern 1pt} \dot{E}_{d}^{rp} + (1 - \omega ){\kern 1pt} {\kern 1pt} {\kern 1pt} \ddot{E}_{d}^{rp} ,} & {(x,y) \ne (i,j)} \\ \end{array} } \right.$$

is called the direction force parameter of $rp$ on direction $d$.

The values of $F_{d}^{lp}$ and $F_{d}^{rp}$ calculated in Eqs. (8) and (9) are normalized as shown in Eq. (10)

10 $$\tilde{F}_{d}^{lp} = \frac{{F_{d}^{lp} - \mathop {\min }\limits_{d} (F_{d}^{lp} )}}{{\mathop {\max }\limits_{d} (F_{d}^{lp} ) - \mathop {\min }\limits_{d} (F_{d}^{lp} )}},\;\tilde{F}_{d}^{rp} = \frac{{F_{d}^{rp} - \mathop {\min }\limits_{d} (F_{d}^{rp} )}}{{\mathop {\max }\limits_{d} (F_{d}^{rp} ) - \mathop {\min }\limits_{d} (F_{d}^{rp} )}}$$

where $\tilde{F}_{d}^{lp}$ and $\tilde{F}_{d}^{rp}$ are normalized values, $\mathop {\max }\limits_{d} (F_{d}^{lp} )$ is the maximum direction force parameter in his or her neighbors of $lp$, $\mathop {\min }\limits_{d} (F_{d}^{lp} )$ is the minimum direction force parameter, $\mathop {\max }\limits_{d} (F_{d}^{rp} )$ is the maximum direction force parameter in his or her neighbors of $rp$, $\mathop {\min }\limits_{d} (F_{d}^{rp} )$ is the minimum direction force parameter.

### Distance parameter

In addition to the direction force parameters affecting pedestrian movement, the exits also have an important impact on pedestrians. Pedestrians will not walk aimlessly in corridors. The purpose of pedestrians is to leave the corridor through the exit. Pedestrians tend to choose the neighborhood closest to the exit as the moving target. Therefore, we use the shortest distance between a cell and an exit to express the attraction of this cell to pedestrians in the CA model. In the scene of bi-directional pedestrian flow, the cell is closer to the left exit, the greater attraction to pedestrians walking to the left and vice versa.

In this paper, the Euclidean distance between a cell and an exit is used as the distance parameter, as shown in Eq. (11)

11 $$D_{c}^{l} = \mathop {\min }\limits_{w} (\sqrt {(x - x_{w}^{l} )^{2} + (y - y_{w}^{l} )^{2} } {\kern 1pt} {\kern 1pt} {\kern 1pt} ),\;D_{c}^{r} = \mathop {\min }\limits_{w} (\sqrt {(x - x_{w}^{r} )^{2} + (y - y_{w}^{r} )^{2} } {\kern 1pt} {\kern 1pt} {\kern 1pt} )$$

where $c$ is for all a cell, $(x,y)$ is the coordinate of $c$, $D_{c}^{l}$ is the shortest distance between $c$ and the left exit, $D_{c}^{r}$ is the shortest distance to the right exit, $(x_{w}^{l} ,y_{w}^{l} )$ is the coordinate of cell $w{\kern 1pt} {\kern 1pt} {\kern 1pt} (w = 1,2, \cdots ,W)$ in the left exit, $(x_{w}^{r} ,y_{w}^{r} )$ is the coordinate of $w$ in the right exit.

The space movement distance is 1 when a pedestrian moves to directly ahead, left, or right cell in a time-step. However, the space movement distance is $\sqrt 2$, when a pedestrian moves to the front left or front right cell. Therefore, If $lp$ is for all a pedestrian moving leftwards, $(i,j)$ is the current position's coordinate, and his or her 6 neighborhood coordinate is $(x,y) \in \{ i - 1,i\} \times \{ j - 1,j,j + 1\}$. Again, if $rp$ is for all a pedestrian moving rightwards, $(i,j)$ is the current position's coordinate, and his or her 6 neighborhood coordinates is $(x,y) \in \{ i,i + 1\} \times \{ j - 1,j,j + 1\}$. The shortest distances of $lp$ or $rp$ moving to 6 neighborhood positions are shown in Eq. (12)

12 $$S_{d}^{lp} = \left\{ {\begin{array}{*{20}l} {0,{\kern 1pt} {\kern 1pt} {\kern 1pt} {\kern 1pt} {\kern 1pt} {\kern 1pt} {\kern 1pt} {\kern 1pt} {\kern 1pt} {\kern 1pt} {\kern 1pt} {\kern 1pt} {\kern 1pt} {\kern 1pt} {\kern 1pt} {\kern 1pt} {\kern 1pt} {\kern 1pt} {\kern 1pt} {\kern 1pt} {\kern 1pt} {\kern 1pt} {\kern 1pt} {\kern 1pt} x = i,y = j} \\ {\frac{{D_{0}^{lp} - D_{1}^{lp} }}{1},x = i,y = j + 1} \\ {\frac{{D_{0}^{lp} - D_{2}^{lp} }}{\sqrt 2 },{\kern 1pt} {\kern 1pt} {\kern 1pt} {\kern 1pt} {\kern 1pt} {\kern 1pt} {\kern 1pt} {\kern 1pt} {\kern 1pt} {\kern 1pt} {\kern 1pt} {\kern 1pt} {\kern 1pt} {\kern 1pt} {\kern 1pt} x = i - 1,y = j + 1} \\ {\frac{{D_{0}^{lp} - D_{3}^{lp} }}{1},{\kern 1pt} {\kern 1pt} {\kern 1pt} x = i - 1,y = j} \\ {\frac{{D_{0}^{lp} - D_{4}^{lp} }}{\sqrt 2 },{\kern 1pt} {\kern 1pt} {\kern 1pt} {\kern 1pt} {\kern 1pt} {\kern 1pt} {\kern 1pt} {\kern 1pt} {\kern 1pt} {\kern 1pt} {\kern 1pt} {\kern 1pt} {\kern 1pt} {\kern 1pt} {\kern 1pt} {\kern 1pt} x = i - 1,y = j - 1} \\ {\frac{{D_{0}^{lp} - D_{5}^{lp} }}{1},{\kern 1pt} {\kern 1pt} {\kern 1pt} {\kern 1pt} x = i,y = j - 1} \\ \end{array} } \right.,\quad S_{d}^{rp} = \left\{ {\begin{array}{*{20}l} {0,{\kern 1pt} {\kern 1pt} {\kern 1pt} {\kern 1pt} {\kern 1pt} {\kern 1pt} {\kern 1pt} {\kern 1pt} {\kern 1pt} {\kern 1pt} {\kern 1pt} {\kern 1pt} {\kern 1pt} {\kern 1pt} {\kern 1pt} {\kern 1pt} {\kern 1pt} {\kern 1pt} {\kern 1pt} {\kern 1pt} {\kern 1pt} x = i,y = j} \\ {\frac{{D_{0}^{rp} - D_{1}^{rp} }}{1},x = i,y = j + 1} \\ {\frac{{D_{0}^{rp} - D_{2}^{rp} }}{\sqrt 2 },{\kern 1pt} {\kern 1pt} {\kern 1pt} {\kern 1pt} {\kern 1pt} {\kern 1pt} {\kern 1pt} {\kern 1pt} x = i + 1,y = j + 1} \\ {\frac{{D_{0}^{rp} - D_{3}^{rp} }}{1},{\kern 1pt} {\kern 1pt} {\kern 1pt} x = i + 1,y = j} \\ {\frac{{D_{0}^{rp} - D_{4}^{rp} }}{\sqrt 2 },{\kern 1pt} {\kern 1pt} {\kern 1pt} {\kern 1pt} {\kern 1pt} {\kern 1pt} {\kern 1pt} {\kern 1pt} {\kern 1pt} {\kern 1pt} {\kern 1pt} {\kern 1pt} {\kern 1pt} {\kern 1pt} x = i + 1,y = j - 1} \\ {\frac{{D_{0}^{rp} - D_{5}^{rp} }}{1},{\kern 1pt} {\kern 1pt} {\kern 1pt} {\kern 1pt} x = i,y = j - 1} \\ \end{array} } \right.$$

where $S_{d}^{lp}$ is the distance parameter from $(i,j)$ to $(x,y)$ in the process of $lp$ moving to the neighbors, and $S_{d}^{rp}$ is the distance parameter to $(x,y)$ in the process of $rp$ movement.

The values of $S_{d}^{lp}$ and $S_{d}^{rp}$ calculated in Eq. (12) are normalized as shown in Eq. (13)

13 $$\tilde{S}_{d}^{lp} = \frac{{S_{d}^{lp} - \mathop {\min }\limits_{d} (S_{d}^{lp} )}}{{\mathop {\max }\limits_{d} (S_{d}^{lp} ) - \mathop {\min }\limits_{d} (S_{d}^{lp} )}},\;\tilde{S}_{d}^{rp} = \frac{{S_{d}^{rp} - \mathop {\min }\limits_{d} (S_{d}^{rp} )}}{{\mathop {\max }\limits_{d} (S_{d}^{rp} ) - \mathop {\min }\limits_{d} (S_{d}^{rp} )}}$$

where $\tilde{S}_{d}^{lp}$ and $\tilde{S}_{d}^{rp}$ are normalized values. $\mathop {\max }\limits_{d} (S_{d}^{lp} )$ is the maximum distance parameter in the process of $lp$ moving to the neighbors, and $\mathop {\min }\limits_{d} (S_{d}^{lp} )$ is the minimum distance parameter. $\mathop {\max }\limits_{d} (S_{d}^{rp} )$ is the maximum distance parameter in the process of $rp$ moving to the neighbors, and $\mathop {\min }\limits_{d} (S_{d}^{rp} )$ is the minimum distance parameter.

### Transition probability

At each time-step $t$, the transition probability of a pedestrian $lp$ moving to the neighbors is

14 $$\begin{aligned} C_{d}^{lp} & = \frac{{N_{d}^{lp} }}{{\sum\limits_{d} {N_{d}^{lp} } }} \\ N_{d}^{lp} & = \exp (\tilde{F}_{d}^{lp} + \tilde{S}_{d}^{lp} ) \\ \end{aligned}$$

where $C_{d}^{lp}$ is the transition probability of $lp$ choosing the *d*-th neighbor ($d = 0,1, \cdots ,5$). $N_{d}^{lp}$ is the attraction value of the *d*-th neighbor to $lp$.

The transition probability of a pedestrian $rp$ moving to the neighbors is

15 $$\begin{aligned} C_{d}^{rp} & = \frac{{N_{d}^{rp} }}{{\sum\limits_{d} {N_{d}^{rp} } }} \\ N_{d}^{rp} & = \exp (\tilde{F}_{d}^{rp} + \tilde{S}_{d}^{rp} ) \\ \end{aligned}$$

where $C_{d}^{rp}$ is the transition probability of $rp$ choosing the *d*-th neighbor. $N_{d}^{rp}$ is the attraction value of the *d*-th neighbor to $rp$.

### Movement rules

The model adopts a synchronous parallel update mechanism. At each time-step $t$, pedestrians decide on the next movement according to the direction force parameter and distance parameter. Each pedestrian follows the following movement rules during the simulation.

At each time step, pedestrians can only choose the target position from the neighborhood as shown in Fig. 14.

- The model calculates the transition probability $C_{d}^{lp}$ of each *lp*'s neighbors and $C_{d}^{rp}$ of every *rp*'s neighbors at each time step.
- When a pedestrian determines the target position, he or she chooses the neighbor with the maximum transition probability. The system randomly selects a neighbor, when there are multiple maximum values.
- When multiple pedestrians choose the same neighbor at a time-step, the system will randomly select a pedestrian to enter this neighborhood, while not being selected pedestrians will wait in the situ in the next time-step.
- If two opposite pedestrians choose each other's positions at the same time, they will exchange their positions in the next time-step.
- When the pedestrian moves to the exit, the system adopts two ways to simulate: (a) this pedestrian will leave the system in the next time-step, (b) if a pedestrian exits through the left exit, he or she will re-enter through the right exit, these two exits have the same *x*-coordinate, meaning they are located on the same horizontal line; and if this pedestrian at the right exit, he or she will re-enter through the left exit.
- Since there are two processing methods for pedestrians to arrive at the exit, the end conditions of the simulation corresponding to each method are: (a) all pedestrians leave the system, and (b) the simulation reaches a steady state.

## Simulation and results

Pedestrians are randomly distributed in the system $\Omega^{2}$. $N$ is the total number of pedestrians. Pedestrian density $K$(%) is defined as the value of $N$ divided by the total number of cells $L \times W$. Moving left and right proportion of pedestrians $P^{l} /P^{r}$ is expressed as the ratio of the total number of moving left pedestrians to the total number of moving right pedestrians. Pedestrian evacuation time $T$(s) is described as the required time of all pedestrians leaving the system. The space moving distance is $\sqrt 2$, when a pedestrian moves to the front left or front right cell in a time-step, but the moving distance to his or her destination is 1. We stipulate that the moving speed to directly ahead, front left or front right cell is 1.0 m/s. The space moving distance is 1 when a pedestrian moves to the left or right cell, but the moving distance to his or her destination is 0. We stipulate that the moving speed to the left, right, or current cell is 0 m/s. Hence, the average speed of pedestrian flow $\overline{V}$(%) is defined as the total number of pedestrians, who have a speed of 1.0 m/s, divided by the value of $N$. The system volume $F$(%) is expressed as the product of pedestrian density $K$ and the average speed of pedestrian flow $\overline{V}$. In the process of the simulation, we take the average value of the operated 10 results as a statistical index to reduce the effect of the initial condition on the statistical indexes.

### Pedestrian flow evacuation simulation

**Figure 17:**
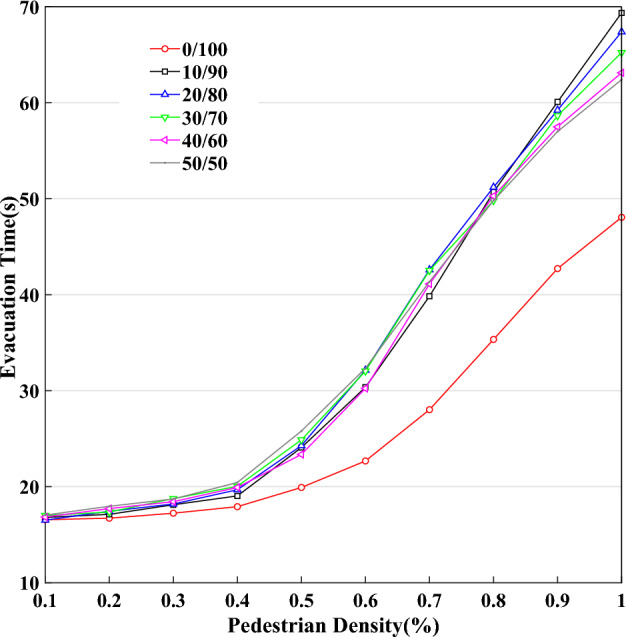
The curves of pedestrian evacuation time change with pedestrian density.

When the simulation chooses the first end condition, this paper studies the curves of pedestrian evacuation time $T$ changing with pedestrian density $K$ as shown in Fig. 17, based on $L \times W = 40 \times 20$, and $P^{l} /P^{r} = 0/100,10/90,20/80,$ $30/70,40/60,50/50$. In the case of fixed system scale, there is a significant difference between unidirectional pedestrian flow and bi-directional pedestrian flow, because the movement of bi-directional pedestrian flow is more complex than unidirectional pedestrian flow. Figure 17 shows that there is an increasing trend in the process of pedestrian evacuation time changing with pedestrian density. There is no significant increasing trend in the condition of the small value $K$. However, there is a significant increasing trend in the condition of the higher value. This difference should have a significant change when pedestrian density approximately equals 0.5. It is demonstrated that $K = 0.{5}$ is a critical value. In the condition of high value $K$, the curves of different $P^{l} /P^{r}$ will have a reversal point. The lower curve will be in above this point because the probability of exchanging position is small between moving left pedestrians and moving right pedestrians in the case of small value $K$, yet the probability of exchanging position is significantly increased in the condition of high value $K$. Hence, a more balanced $P^{l} /P^{r}$ of bi-directional pedestrian flow should have a higher probability of exchanging position and the average speed.

Pedestrian evacuation time is approximately linear increasing trend changing with pedestrian density in the traditional model of CA. However, the model in this paper shows that pedestrian evacuation time is approximately exponentially increasing trend changing with pedestrian density, this result meets the actual circus better, more external, and easier to understand. When the pedestrian density reaches a critical value, pedestrians begin to feel crowded, and the average speed will decrease rapidly, which should lead evacuation time to increase significantly.

**Figure 18:**
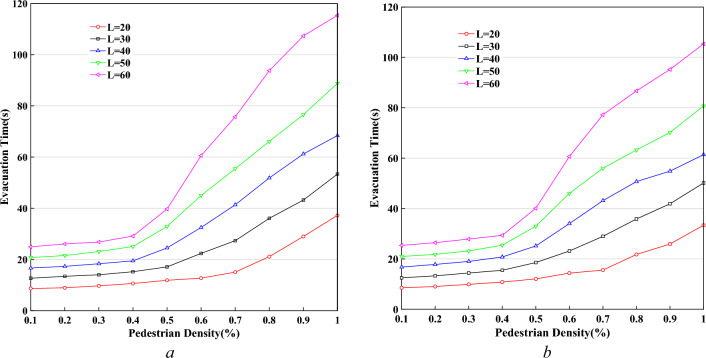
The curves of pedestrian evacuation time based on different lengths of the system. (**a**) $P^{l} /P^{r} = {\kern 1pt} {20/80}$, (**b**) $P^{l} /P^{r} = {\kern 1pt} {\kern 1pt} {50/50}$.

Figure 18 shows the curves of pedestrian evacuation time $T$ changing with pedestrian density $K$, based on different lengths of the system, $W = 20$ and $P^{l} /P^{r} = 20/80,{\kern 1pt} 50/50$. In the case of fixed system width, the pedestrian evacuation time has a significant difference in the condition of different system lengths. It is shown that the length is a key factor in pedestrian evacuation time.

**Figure 19:**
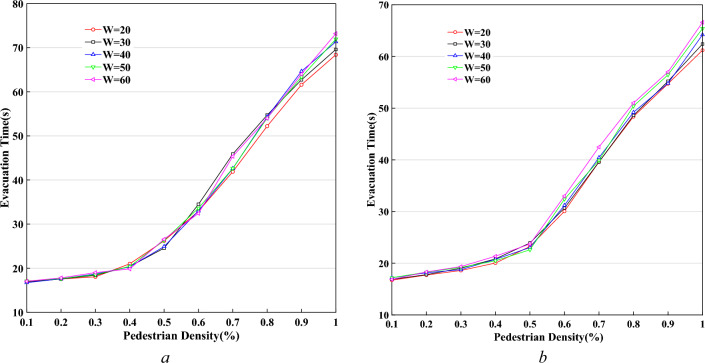
The curves of pedestrian evacuation time based on different widths of the system. (**a**) $P^{l} /P^{r} = {\kern 1pt} {20/80}$, (**b**) $P^{l} /P^{r} = {\kern 1pt} {\kern 1pt} {50/50}$.

Figure 19 demonstrates the curves of pedestrian evacuation time $$T$$ changing with pedestrian density $$K$$, based on different widths of the system, $$L = 40$$, and $$P^{l} /P^{r} = 20/80,$$$$50/50$$. In the case of fixed system length, there is no significant difference in the condition of different system width. It is expressed that expanding the system width is an effective way to control pedestrian evacuation time in the case of big gatherings.

### Pedestrian flow steady-state simulation

**Figure 20:**
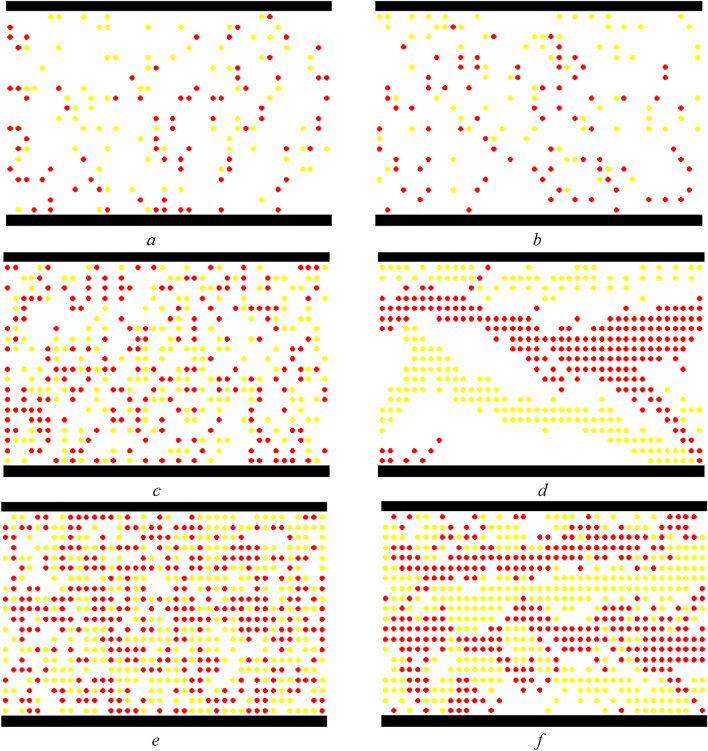
The status of bi-directional pedestrian flow in different pedestrian densities during the process of simulation. (**a**) Initial status ($K = 0.2$), (**b**) the status of strolling flow ($K = 0.2$), (**c**) initial status ($K = 0.5$), (**d**) the status of flows in directional separated lanes ($K = 0.{5}$), (**e**) initial status ($K = 0.{8}$), (**f**) the status of dynamic multi-lane flow ($K = 0.{8}$).

When the simulation selects the second end condition, this paper analyzes the process and evolution of pedestrian movement as demonstrated in Fig. 20, based on $L \times W = 40 \times 20$, $K = 0.2,0.5,0.8$, and $P^{l} /P^{r} = 50/50$. In the condition of $K = 0.2$, Fig. 20a shows the initial status, and Fig. 20b demonstrates the status of the strolling flow when the system is running to a steady state. Despite the impact of the direction force parameter, pedestrians are still strolling in the system when pedestrian density is small. In this case of $K = 0.5$, Fig. 20c shows the initial status, and Fig. 20d demonstrates the status of flows in directional separated lanes. There is an appearance to separate bi-directional pedestrians when pedestrian density is a moderate value. In the circumstance of $K = 0.8$, Fig. 20e shows the initial status, and Fig. 20f demonstrates the status of dynamic multi-lane flow. The pedestrian movement in one direction should form a line when pedestrian density is high. These experimental results agree well with Blue's studies^3^. The status of bi-directional pedestrian flow is changing in the process of movement because every pedestrian should choose the target position based on his or her psychological characteristics and the circumstances surrounding it on a microscopic level. Therefore, it is expressed the self-organization phenomena on a macroscopic level.

**Figure 21:**
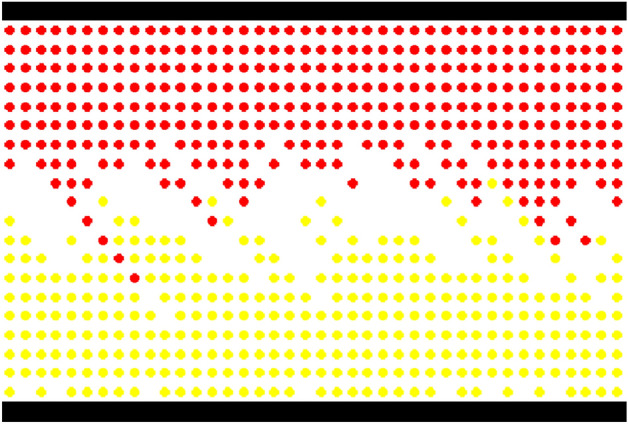
The status of bi-directional pedestrian flow under the pedestrian "right-leaning" rule ($K = 0.{8}$).

Figure 20f shows that bi-directional pedestrian flow appears as dynamic multi-lane flow when the pedestrian density is high. Although the dynamic multi-lane flow in the same direction can improve pedestrian evacuation efficiency, it still cannot effectively reduce the opposite conflict between the bi-directional pedestrian flow. Therefore, the pedestrian "right-leaning" rule is introduced into the model, that is, the pedestrians tend to choose to move toward the front or right cell in the target position selection. Only when the front and right cells are occupied, the pedestrian will choose the left cell. In the circumstance of $L \times W = 40 \times 20$, $P^{l} /P^{r} = 50/50$, and $K = 0.8$, Fig. 21 demonstrates the pedestrian flow state in the simulation process when pedestrians adopt the "right-leaning" rule. As can be seen from Fig. 21, the bi-directional pedestrian flow quickly realizes the stratification under the action of the "right-leaning" rule and effectively avoids the opposite conflict of pedestrians. However, if all pedestrians adopt the "right-leaning" rule, pedestrians will be excessively concentrated near the wall; it will increase the risk of pedestrian evacuation in this area. Therefore, the moderate introduction of the pedestrian "right-leaning" rule cannot only effectively avoid the opposite conflict of pedestrians, but also reduce the pressure of pedestrian evacuation near the wall to a certain extent.

**Figure 22:**
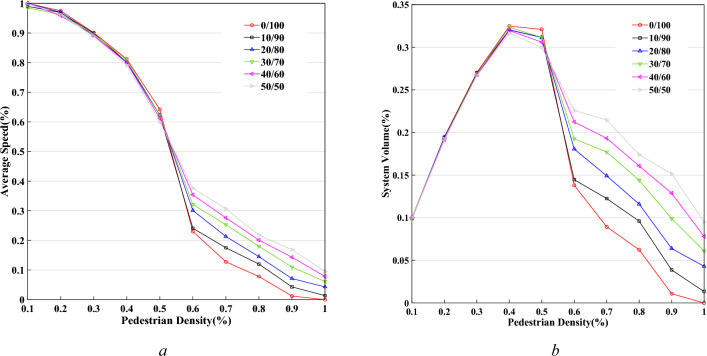
The curves of average speed and system volume change with pedestrian density. (**a**) The density-speed curve, (**b**) the density-volume curve.

The paper further analyses the curves of the average speed $$\overline{V}$$ and system volume $$F$$ changing with pedestrian density $$K$$ as demonstrated in Fig. 22, based on $$L \times W$$$${ = }40 \times 20$$, and $$P^{l} /P^{r} = 0/100,10/90,20/80,30/70,40/60,50/50$$. It is shown that there is a decreasing trend in the process of the average speed changing with pedestrian density. There is a slow decline in the case of small value $$K$$. However, there is a significant decline in the condition of higher value. This difference will have a significant change when pedestrian density approximately equals 0.5. This demonstrates that there should be a certain degree of congestion in the process of pedestrian movement when pedestrian density exceeds this critical value. Influenced by the average speed, the system volume has an increasing trend in the process of changing with pedestrian density based on small value $$K$$. There is a significant decline in the case of pedestrian density exceeding this critical value.

In the process of simulation, pedestrian density $K$ is defined as the total number of pedestrians divided by the total cell number in the system. This is different from the actual pedestrian flow density. Consequently, pedestrian density $K$ is needed to convert before the comparison between the experimental results and the realistic pedestrian flow characteristics. When pedestrian density $K$ equals 0.5 in the model, the actual pedestrian flow density is 3.125 ped/m^2^ based on the size of a cell corresponding to 0.4 m × 0.4 m. The average speed of pedestrian flow will have a significant decline when pedestrian density is about 3.125 ped/m^2^. For unidirectional pedestrian flow, the average speed is close to zero when pedestrian density $K$ approximately equals 0.9 (5.625 ped/m^2^). This experimental result agrees well with Tregenza 's study^27^ which observed the real pedestrian flow. It is shown that pedestrians begin to drag and move slowly in the process of movement when pedestrian density is more than 3.125 ped/m^2^, so the average speed begins to decline significantly. When pedestrian density is more than 5.625 ped/m^2^, the average speed is close to zero. For bi-directional pedestrian flow, the average speed equals zero in the circumstance of high pedestrian density based on the traditional CA model. However, the model in this paper is shown that the average speed is a small value, but is not zero. And a more balanced $P^{l} /P^{r}$ of bi-directional pedestrian flow has a relatively high average speed. Because the probability of exchanging position has a significant increase in the condition of a high pedestrian density, a more balanced $P^{l} /P^{r}$ will have a higher probability and average speed. But overall, the values of average speed and system volume are very small when pedestrian density is more than 5.625 ped/m^2^, and bi-directional pedestrian flow is in a very congested state at this time. This result is more consistent with the observation of real pedestrian flow.

## Conclusion

This paper redefines pedestrian personal space and information processing space based on the direction fuzzy visual field (DFVF). The *k*-nearest neighbor rule constructs the topology of the pedestrian interaction force field. It uses the direction force parameter and distance parameter in DFVF to establish the modified bi-directional pedestrian flow CA model. It analyses the relationship between system scale, evacuation time, pedestrian density, average speed, and system volume, and studies the self-organization phenomenon of bi-directional pedestrian flow emerging in the simulation process. The results show that:

- Evacuation time increases exponentially with pedestrian density. System length is the key factor impacting evacuation time. Appropriately increasing system width is an effective way to reduce evacuation time.
- As the actual pedestrian flow density rises beyond the critical point of around 3.125 ped/m^2^, the average speed starts to drop markedly with any further increase in density. This indicates conspicuous congestion emerges in bi-directional pedestrian flow when density surpasses this critical threshold.
- Under varying pedestrian densities, bi-directional pedestrian flow exhibits three states: strolling flow, directional separated lane flow, and dynamic multi-lane flow.
- The moderate introduction of a pedestrian "right-leaning" rule can not only effectively avoid opposite pedestrian conflicts, but also relieve evacuation pressure near walls to some extent.

The proposed model is beneficial to simulate the diversity in pedestrians' psychological characteristics and would work as a supplement to the theory of bi-directional pedestrian flow. This study provides valuable insights for pedestrian facility design and optimizing pedestrian flow organization.

### Ethical declaration

The study was performed according to the Declaration of Helsinki. The experimental protocols were approved by the Gansu Provincial Department of Transportation, the Gansu Provincial Department of Science and Technology, and the Academic Ethic Committee of Lanzhou Jiaotong University. Informed consent was obtained from all participants before they took part in the survey. This study obtains, stores, manages, interprets, analyzes, and applies data in a manner consistent with ethical standards and social responsibility. The questionnaire survey is conducted for academic research purposes only. The survey aims to collect data about people's opinions on the topics related to the "Direction Fuzzy Visual Field". Participation in this survey is voluntary and anonymous. Informed consent has been obtained from all participants before they take part in the survey. No personally identifiable information will be collected from the participants. The data obtained will be kept confidential and used only for this study. Participants have the right to skip or refuse to answer any question. There are no negative consequences for refusal or withdrawal from participation. The data collected will be anonymously analyzed and used only for the stated research purpose. The participants will not be contacted again in the future. The researchers will make every effort to maintain the anonymity and confidentiality of the participants' data and ensure that it is kept secure.

## Data Availability

The datasets used and/or analyzed during the current study are available from the corresponding author upon reasonable request.
